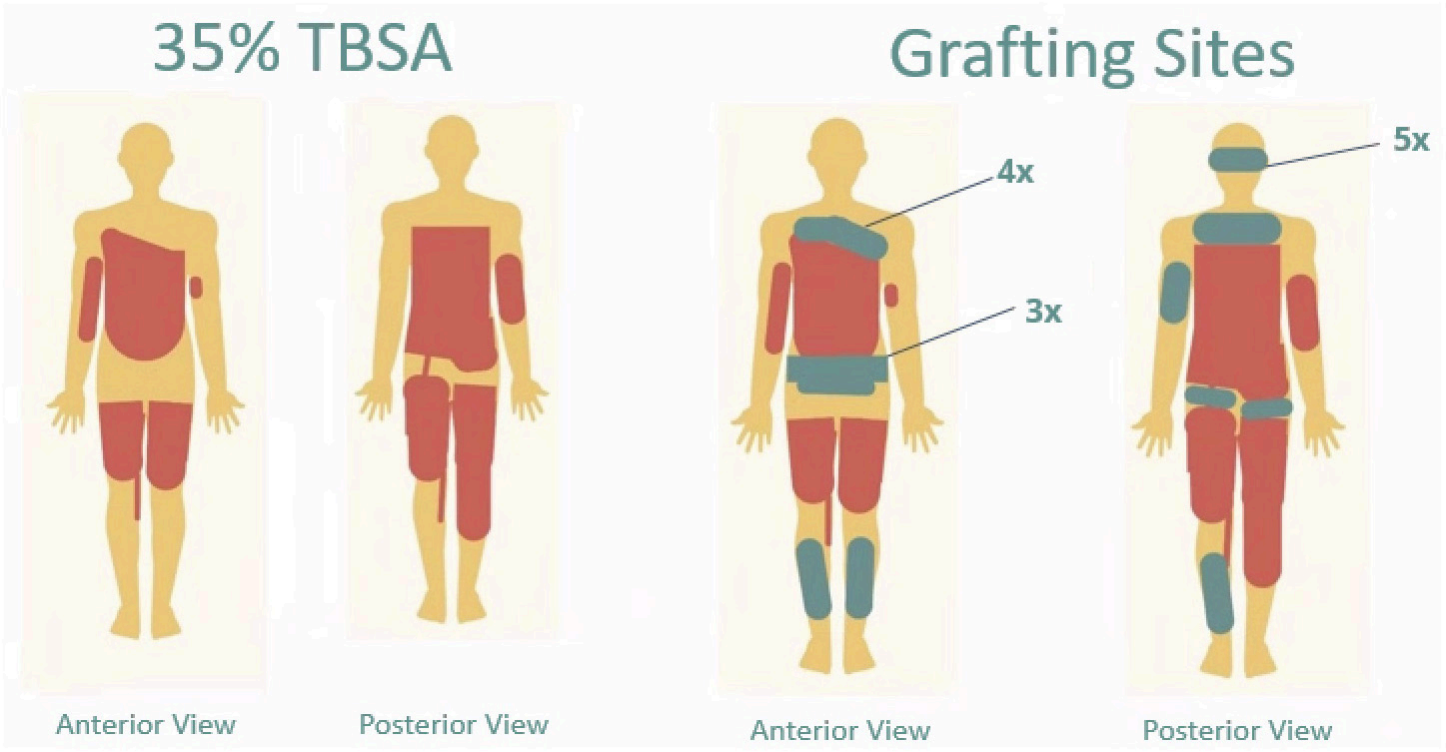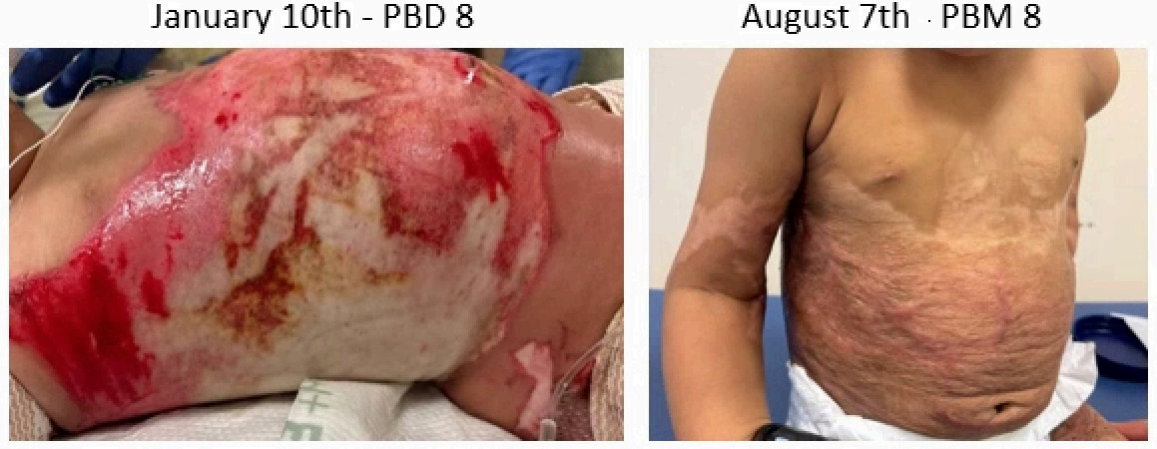# 1001 Intensive OT/PT for a Major Burn in a Toddler at a Low Volume Burn Center

**DOI:** 10.1093/jbcr/iraf019.532

**Published:** 2025-04-01

**Authors:** Colleen Cox, Amber Shojaie

**Affiliations:** Children’s National Trauma and Burn Surgery; Children’s National Trauma and Burn Surgery

## Abstract

**Introduction:**

As a low volume pediatric burn center with few critical major burns, it is pertinent to establish feasible and necessary components to progress patient recovery within the confines of resources. A time intensive therapy plan of care, creative use of therapeutic equipment and environment, and caregiver involvement are necessary components of a successful rehabilitation for a pediatric major burn.

**Methods:**

This case study explores the acute care stay of a two-year-old male with 35% total body surface area deep partial thickness and full thickness scald burns to circumferential torso, circumferential bilateral thighs, buttocks, and bilateral upper extremities from hot water. The patient’s hospital course was complicated by poorly responsive fluid overload with subsequent development of acute respiratory distress syndrome and candidemia bacteremia. Daily occupational and physical therapy services were implemented in the 98-day length of stay. Interventions included individualized positioning guidelines, varied equipment use, splinting, compression, and intentional caregiver involvement. The outcomes being assessed included independence with functional mobility, participation in play, active/passive range of motion, presence of contractures, and hypertrophic scarring. Following discharge, this patient’s medical record was closely reviewed to collect quantitative and qualitative data on the therapy plan of care and subsequent outcomes.

**Results:**

The patient received twice daily therapy starting day three of admission in the intensive care unit and was followed through discharge for 3,702 minutes of combined therapy services. Upon the four-month post-discharge follow-up, the two-year-old patient demonstrated independence with functional mobility, strong participation in play with peers, full active range of motion globally, and a Vancouver Scar Scale score range of six to eight.

**Conclusions:**

In conclusion, early and intensive occupational and physical therapy services for a toddler with a major burn at a low volume burn center is imperative for functional recovery.

**Applicability of Research to Practice:**

This case study provides quantitative and qualitative data to develop a model of therapy that can be implemented for future similar cases at a low volume burn center. Secondarily, this case study demonstrates the effectiveness of increased duration therapy services to insurance agencies and hospital administration.

**Funding for the Study:**

N/A